# Energy imbalance gap was associated with body mass index and sex in Latin American adolescents—results from the ELANS study

**DOI:** 10.3389/fnut.2024.1380315

**Published:** 2024-03-14

**Authors:** Pablo Hernandez, Marianella Herrera-Cuenca, Gerson Ferrari, Rafaela Yépez Almeida, Martha Cecilia Yépez García, Mónica Villar Cáceres, Lilia Yadira Cortés Sanabria, Yaritza Sifontes, Maritza Landaeta-Jimenez, Georgina Gómez, Rafael Monge-Rojas, Rossina G. Pareja, Attilio Rigotti, Irina Kovalskys, Mauro Fisberg

**Affiliations:** ^1^Escuela de Nutrición y Dietética, Facultad de Medicina, Universidad Central de Venezuela, Caracas, Venezuela; ^2^Centro de Estudios del Desarrollo, Universidad Central de Venezuela (CENDES-UCV), Caracas, Venezuela; ^3^Fundación Bengoa para la Alimentación y Nutrición, Caracas, Venezuela; ^4^Department of Nutrition and Health Studies, Framingham University, Framingham, MA, United States; ^5^Department of Nutrition, Simmons University, Boston, MA, United States; ^6^Escuela de Ciencias de la Actividad Física, el Deporte y la Salud, Universidad de Santiago de Chile (USACH), Santiago, Chile; ^7^Facultad de Ciencias de la Salud, Universidad Autónoma de Chile, Providencia, Chile; ^8^Colegio de Ciencias de la Salud, Universidad San Francisco de Quito, Quito, Ecuador; ^9^Departamento de Nutrición y Bioquímica, Pontificia Universidad Javeriana, Bogotá, Colombia; ^10^Departamento de Bioquímica, Escuela de Medicina, Universidad de Costa Rica, San José, Costa Rica; ^11^Instituto Costarricense de Investigación y Enseñanza en Nutrición y Salud (INCIENSA), Cartago, Costa Rica; ^12^Instituto de Investigación Nutricional, La Molina, Lima, Peru; ^13^Centro de Nutrición Molecular y Enfermedades Crónicas, Departamento de Nutrición, Diabetes y Metabolismo, Escuela de Medicina, Pontificia Universidad Católica, Santiago, Chile; ^14^Carrera de Nutrición, Facultad de Ciencias Médicas, Pontificia Universidad Católica Argentina, Buenos Aires, Argentina; ^15^Centro de Excelencia em Nutrição e Dificuldades Alimentaes (CENDA), Instituto Pensi, Fundação José Luiz Egydio Setubal, Hospital Infantil Sabará, São Paulo, Brazil; ^16^Departamento de Pediatria, Universidade Federal de São Paulo, São Paulo, Brazil

**Keywords:** energy balance, energy expenditure, energy intake, energy imbalance gap, underweight, overweight, Latin America

## Abstract

**Introduction:**

Energy imbalance gap (EIG) is defined as the average daily difference between energy intake (EI) and energy expenditure (EE). This study aimed to examine the associations between EIG and sociodemographic and anthropometric variables in the adolescent population of eight Latin America countries.

**Methods:**

A total of 680 adolescents aged 15 to 18 were included in this study. The estimation of EI was based on two non-consecutive 24-h dietary recalls. EE was predicted from Schofield equations using physical activity level obtained through the long version of the International Physical Activity Questionnaire. Sociodemographic data and anthropometric measurements were also obtained. A descriptive analysis and multilevel linear regression models were used to examine associations between variables.

**Results:**

The mean EI, EE, and EIG were 2091.3 kcal, 2067.8 kcal, and 23.5 kcal, respectively. Argentina had the highest EI and EIG, whereas Chile had the lowest EI and EIG. Males had a higher EI (2262.4 kcal) and EE (2172.2 kcal) than females (1930.1 kcal and 2084.5 kcal), respectively (*p* < 0.05). Overweight subjects had a lower EIG than did underweight and normal-weight subjects (*p* < 0.05). Subjects with low socioeconomic status (SES) had a lower EE (2047.0 kcal) than those with a high SES (2164.2 kcal) (*p* < 0.05).

**Conclusion:**

Sex and BMI were associated with EIG in adolescents from Latin America.

## 1 Introduction

Latin America (LA) is a region with several and complex health concern and the double burden of malnutrition is an important one. Globally, there has been an interest in adolescents as a group that is relevant for the future health of the population; thus, the World Health Organization (WHO) has included it among its prioritizing groups (1). A previous global report (2) established that overweight adolescents are increasing in LA, with a prevalence between 15 and 25% in most countries of the region. In contrast, the prevalence of moderate and severe underweight was below 10% for both girls and boys in all LA countries (2). On the other hand, a recent report (3) indicated that in both the LA and the Caribbean, approximately two-thirds of teenagers have insufficient physical activity. More than 40% of the participants were sedentary and more than 20% were completely inactive. These results were more frequent among girls than among boys. These constitute low energy expenditure (EE) indicators in adolescents in the region.

For around a hundred years, it has been thought that weight management is the result of small, persistent differences in energy intake (EI) and EE. The energy imbalance gap (EIG) is defined as the average daily difference between EI and EE (4). Therefore, a positive energy imbalance gap arises when EI is greater than EE, whereas a negative energy imbalance gap occurs when EE exceeds EI (5) by applying the First Law of Thermodynamics (energy conservation principle) (6). This approach is simple and easy to remember by the general population; therefore, it is commonly used for educational purposes in public health, especially in dietary guidelines (7, 8), and it represents a driver for the changes observed in BMI of overweight and obese individuals, and allow to follow epidemiologic trends within a given population (9).

Nevertheless, at the individual level, the energy balance model states that the brain serves as the primary organ responsible for regulating body weight through the integration of neuroendocrine-gastrointestinal signaling pathways that either increase or decrease overall EI (10). The current better understanding of the neuro-endocrine axis where environmental signals are to be integrated and translated into neuro-hormonal signals in the short term that regulate food intake via the hypothalamus, basal ganglia, through the release of ghrelin, peptide YY and GLP-1 and controls the appetite and satiety cycles (10), as demanded by external environmental stimulus including rewards cycle, have allowed to introduce the knowledge to accept the complexity of food intake and its impairments and consequences in the long term including leptin alterations that will run the changes over time, thus impacting the energy balance of an individual.

Additionally, environmental, economic, and social trends, which are external drivers and because of the behavior toward foods, excessive or inadequate intake, significantly impact EIG (11) and therefore translate into all these neuro-endocrine events, making the environmental factors important elements in the regulation of the food intake (10).

Owing to this complex system, an individual’s energy balance can vary daily. Therefore, accurate measures of energy balance in humans are controversial. More precise methods are costly and challenging to perform in public health and epidemiological studies. Nevertheless, knowing the trends within a population is key to monitoring well-being and contributing to the prevention of chronic diseases (12).

The disparity between EI and EE, represents a significant global health challenge. Studies show that exceeding energy needs even by a small amount over time can significantly increase the risk of developing chronic diseases, including obesity, cardiovascular disease, type 2 diabetes, and certain cancers (13, 14). On the other hand, when there is an insufficient intake of energy compared to energy expenditure, it can lead to malnutrition and deficiencies in essential nutrients (15). This could weaken the immune system, impair growth and development, and increase the risk of infections and other health problems (16). Furthermore, energy imbalance can also impact mental health and well-being. Poor diet and lack of physical activity could contribute to feelings of fatigue, low mood, and decreased cognitive function (15). These factors can further exacerbate the risk of developing mental health disorders such as depression, anxiety and eating disorders, especially in adolescents (17).

To date, only a limited number of studies have examined EIG in a large population (4, 5, 9, 18), finding heterogeneity in EIG by ethnicity, sex, and body mass index (BMI) in adults. Fallah-Fini et al. (4) quantified the dynamics of the EIG among New Zealand adults over 3 decades using a new population-level system dynamics model. They found that there was an inconsistent pattern over time, especially in different ethnic, sex, and BMI subpopulations, i.e., higher BMI was associated with both a higher and a lower EIG. In Japanese adult population (18), the trend of EIG was studied by near 4 decades. They found that EIG was associated to sex and weight groups in this population. The EIG for men ranged from −3.5 kcal/day for a BMI of ≥30.0 kg/m^2^ to 4.6 kcal/day for a BMI of 15.0 to 17.9 kg/m^2^. In a Belgium population, Fallah-Fini et al. (9) found that there was some heterogeneity in the patterns by BMI class, and no consistent patterns emerged over time. These studies, while valuable, do not include data from the Latin American population. Additionally, they are longitudinal rather than cross-sectional studies, which limits their applicability for direct comparison of results. On the other hand, the authors’ previous experience with adults in LA (5), found that overall EIG was positive, meaning people consumed more energy than they were expended. This was more pronounced in men and people with higher socioeconomic status. People who were overweight or obese in Argentina, Costa Rica, Ecuador, Peru, and Venezuela had a significantly lower EIG than those who were underweight. These findings suggest high variability in the EIG and its correlates in the eight LA countries. For the age group of adolescents, much uncertainty still exists about the relationship between EIG and socioeconomic status, anthropometrics, and lifestyle.

This context highlights the relevance of studying the characteristics associated with maintaining a good balance between EI and EE in adolescents as a key component of overall regional prevention policies. Being overweight in adolescents can cause various health and emotional problems including hypertension, dyslipidemia, type 2 diabetes, metabolic syndrome, obstructive sleep apnea, eating disorders, and depression (19, 20). However, undernutrition can also increase the risk of other chronic health problems such as insulin resistance, lowered fat oxidation, increased risk of diabetes in adulthood, reduced energy expenditure, dyslipidemia, hypertension, and diminished manual worker capacity (21).

This study aimed to examine the associations between the EIG and sociodemographic and anthropometric variables in the adolescent population of the eight LA countries evaluated.

## 2 Materials and methods

### 2.1 Research design and participants

The Latin American Study of Nutrition and Health (Estudio Lationamericano de Nutrición y Salud; ELANS) is a multicenter cross-sectional nutritional and health surveillance study of a nationally representative sample of urban populations from eight Latin American countries (Argentina, Brazil, Chile, Colombia, Costa Rica, Ecuador, Peru, and Venezuela). This study addressed EI, EE, and anthropometric data in 10,134 individuals ranging in age from 15 to 65 years, in a lapse between September 2014 and July 2015.

A multistage sampling process was used to select the sample, stratified by geographical location, sex, age, and socioeconomic status (SES), with a random selection of primary and secondary sampling units for the urban population in order to achieve an urban representative sample (22).

In this study, adolescents were considered to be between 15 and 18 years of age. This was because the minimum interview age for the ELANS study was 15 years and, for this study, after 18 years of age, the subjects were considered adults, and informed consent was no longer requested from parents. Healthy adolescents from the urban areas of each country were selected as participants. Excluded from the study were adolescents without parental or legal guardian consent, pregnant or lactating women, individuals residing in non-household settings like hospitals, regiments, and nursing homes, individuals with significant physical or mental impairments, such as musculoskeletal diseases, recent surgeries, severe asthma, dementia, major depression, and those unable to read. These subjects were excluded because their daily intake may be limited by the disability or the care of responsible persons, physical limitations may make body measurements difficult, and illiterates may have difficulty understanding survey instructions and forms, which could lead to errors in the data.

**Figure 1:**
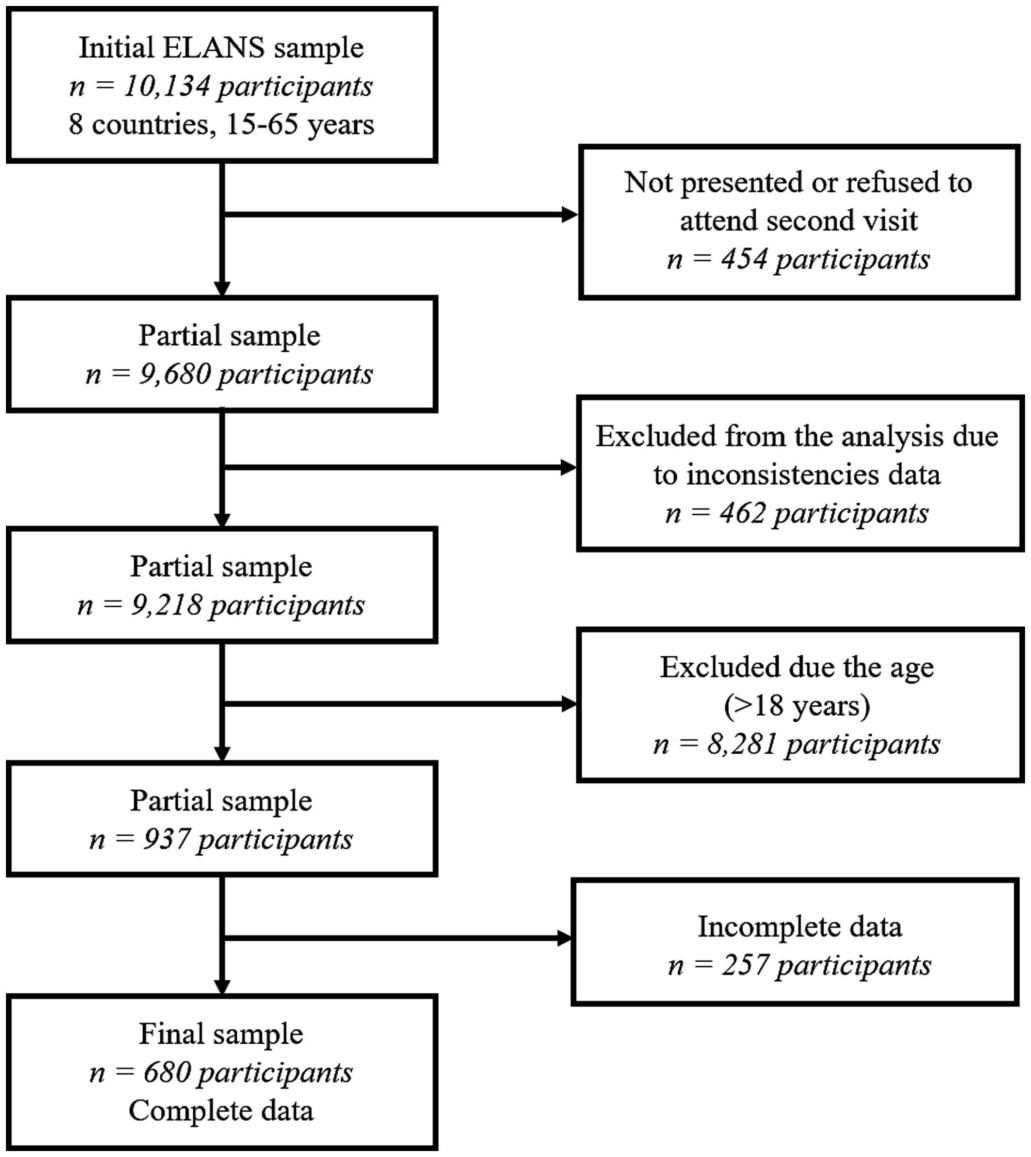
Selection process flowchart of adolescent participants.

Of the partial sample of 9,680 participants, 462 were excluded because of inconsistencies, including duplicate observations or observations that did not pass the supervisor’s quality check. Of the 9,218 subjects, only 937 were adolescents (15–18 years), and another 257 were excluded due to incomplete data. The final sample comprised 680 adolescents (Figure 1).

Additional details of the study design and protocol have been described elsewhere (22, 23). All parents or patient representatives signed informed consent forms. The Western Institutional Review Board (#20140605) approved the study protocol and was registered on the Clinical Trials website (#NCT02226627).

### 2.2 Socioeconomic and anthropometric variables

A sociodemographic questionnaire was developed to evaluate the sociodemographic characteristics of adolescents, such as country, sex, age, SES, educational level, and ethnicity. Household SES was measured using appropriate scales for each country and was classified as high, middle, or low. For educational level, the categories were basic, high school, and undergraduate. Ethnicity was obtained based on self-reported race or ethnicity and divided into five groups: Caucasian, Mestizo, Afro-American, Indigenous and Others.

After administering the questionnaire, adolescents’ body weight and height were measured and recorded following the standard method, wearing light clothing and no shoes. The weight (kg) of the participants was determined using an electronic scale (Seca^®^, Hamburg, Germany) with a measurement precision of 0.1 kg. Height (cm) was measured using a portable stadiometer (Seca^®^, Hamburg, Germany) with an accuracy of 0.1 cm. Body Mass Index (BMI) was calculated by dividing the weight in kilograms by the height in meters squared. The BMI for age and height for age were assessed based on the reference values provided by the WHO (24) as body mass index z-score (zBMI) and height-for-age z-score (HAZ), which was designed as a universal norm and takes into consideration the widely differing ethnic backgrounds and cultural settings (25). These indicators were classified as underweight (zBMI <−2), normal weight (zBMI ≥ −2 to ≤ +1), or overweight (zBMI > +1). Owing to the low frequency of obesity (zBMI > +2), it was grouped together in one category with “overweight.” On the other hand, height was classified as low height (HAZ < −2), risk of low height (HAZ ≥ −2 to < −1), and normal height (HAZ ≥ −1). In this work, a classification for the risk of low height was also carried out, due to the current trend toward diagnosing not only established stunting, but also the risks of developing this nutritional privation, in accordance with local guidelines and references (26, 27), and the relevance of taking the window of opportunity for interventions in adolescents at risk.

### 2.3 Energy intake

A dietary assessment that involved two non-consecutive 24-h food recalls was used to estimate the EI. The Multiple Pass Method was used to evaluate all foods and beverages consumed during the previous day (28). To estimate the portion sizes, photographic albums of common foods from each country and household utensils were used. The selected portions were transformed into grams or milliliters by a group of trained nutritionists.

In another step, macronutrients consumption was transformed into EI using the software Nutrition Data System for Research (NDS-R, Minnesota University, MN, United States). Version 2013. The Multiple Source Method (MSM), a web-based statistical modeling technique proposed by the European Prospective Investigation into Cancer and Nutrition (EPIC) (29), was employed to determine the typical daily EI, taking into account individual variations.

### 2.4 Energy expenditure

The EE was determined by multiplying the Basal Metabolic Rate (BMR) with Physical Activity (PA) utilizing the following formula:


EE=BMR×PA

To estimate the BMR, the equations of the FAO/WHO report “Human Energy Requirements” (30) and the activity factors for physical activity (PA) published by Gerrior et al. (31) were used, which comprised variables such as sex, age, and weight.

PA was assessed using the International Physical Activity Questionnaire (IPAQ) in its extended Spanish version, which was validated for LA (32). The active transportation and leisure-time physical activity domains were incorporated into the study because of their higher significance in informing public health policies and programs (32) and the limited reliability of the IPAQ questions related to occupational and home-based physical activity among adolescents in LA countries.

Therefore, the data collected from the questionnaire regarding physical activity (PA) were expressed as the amount of time spent walking, moderate activity, and vigorous activity per day. The Compendium of Physical Activities was used to determine the metabolic equivalents (METs) for each physical activity, which were expressed as minutes/day and minutes/week (MET-min/day and MET-min/week, respectively) (33). Finally, the IPAQ protocol was used to classify participants into three categories: high, moderate, or low physical activity levels. The IPAQ has been validated through objective methods, i.e., accelerometer (model GT1M) to evaluate PA in adolescents from different countries, with correlations ranging from 0.20 to 0.29 (34).

### 2.5 Energy imbalance gap

The EIG was defined as the average daily difference between EI and EE, using the formula:


EIG=EI−EE

Thus, if a negative number is obtained, it is because the EE exceeds the EI, whereas if the EI is above the EE, a positive number is obtained (5, 18).

### 2.6 Statistical analysis

The Kolmogorov–Smirnov test was used to confirm whether the data adhered to a normal distribution. The means, 95% confidence intervals (95% CI), specific percentiles (3rd, 10th, 25th, 50th, 75th, 90th, and 97th percentiles), and percentages were calculated as required to describe the variables. The weighting process was carried out based on sociodemographic characteristics such as sex, SES, and country.

Multilevel linear regression models (with b coefficients and 95% confidence intervals) were employed to investigate the associations between sociodemographic characteristics (acting as independent variables) and EI, EE, and EIG (functioning as dependent variables) in each country and collectively. These models satisfied the assumptions of linearity, independence, homoscedasticity, and normality and included regions and cities as random effects. Furthermore, they were adjusted for sex, SES, education level, ethnicity, and BMI and provided unstandardized beta coefficients and 95% confidence intervals. A significance level of 5% was considered to be statistically significant.

All statistical analyses were conducted using IBM SPSS software version 26 (IBM Corp., Armonk, New York, United States).

## 3 Results

The total sample comprised 680 adolescents (mean age 16.5 ± 1.2 years). The features of the participants are listed in Table 1, which provides a comprehensive overview of their characteristics. Overall, the proportion of males was higher than that of females, except in Venezuela. About half (53.1%) were classified as having a low SES, 82.8% had a basic or lower educational level, and 48.7% were of the Mestizo ethnicity. More than 80% of the adolescents had normal BMI and height for age. The prevalence of low height was higher than the prevalence of overweight in this group of adolescents (7.5% vs. 6.2%, respectively).

**Table 1:** Distribution of adolescents according to sociodemographic variables by country.

Variables n (%)	Argentina	Brazil	Chile	Colombia	Costa Rica	Ecuador	Peru	Venezuela	Overall
	87 (12.8)	123 (18.1)	64 (9.4)	73 (10.7)	69 (10.2)	73 (10.7)	97 (14.3)	94 (13.8)	680 (100.0)
Sex									
Male	57 (65.5)	76 (61.8)	35 (54.7)	43 (58.9)	42 (60.9)	41 (56.2)	51 (52.6)	45 (47.9)	390 (57.4)
Female	30 (34.5)	47 (38.2)	29 (45.3)	30 (41.1)	27 (39.1)	32 (43.8)	46 (47.4)	49 (52.1)	290 (42.6)
Socio-economic status									
Low	55 (63.2)	48 (39.0)	25 (39.1)	49 (67.1)	23 (33.3)	34 (46.6)	50 (51.5)	77 (81.9)	361 (53.1)
Middle	29 (33.3)	64 (52.0)	32 (50.0)	19 (26.0)	38 (55.1)	32 (43.8)	31 (32.0)	13 (13.8)	258 (37.9)
High	3 (3.4)	11 (8.9)	7 (10.9)	5 (6.8)	8 (11.6)	7 (9.6)	16 (16.5)	4 (4.3)	61 (9.0)
Educational level									
Basic	83 (95.4)	99 (80.5)	60 (93.8)	56 (76.7)	65 (94.2)	71 (97.3)	52 (53.6)	77 (81.9)	563 (82.8)
High school	3 (3.4)	24 (19.5)	3 (4.7)	15 (20.5)	4 (5.8)	2 (2.7)	44 (45.4)	3 (3.2)	98 (14.4)
Undergraduate	1 (1.1)	0 (0.0)	1 (1.6)	2 (2.7)	0 (0.0)	0 (0.0)	1 (1.0)	14 (14.9)	19 (2.8)
Ethnicity									
Caucasian	59 (67.8)	49 (39.8)	24 (37.5)	25 (34.2)	36 (52.2)	4 (5.5)	7 (7.2)	30 (31.9)	234 (34.4)
Mestizo	16 (18.4)	22 (17.9)	31 (48.4)	38 (52.1)	19 (27.5)	65 (89.0)	85 (87.6)	55 (58.5)	331 (48.7)
Afro-American	1 (1.1)	23 (18.7)	0 (0.0)	2 (2.7)	0 (0.0)	3 (4.1)	1 (1.0)	2 (2.1)	32 (4.7)
Indigenous	0 (0.0)	4 (3.3)	2 (3.1)	2 (2.7)	1 (1.4)	0 (0.0)	0 (0.0)	0 (0.0)	9 (1.3)
Others	11 (12.6)	25 (20.3)	7 (10.9)	6 (8.2)	13 (18.8)	1 (1.4)	4 (4.1)	7 (7.4)	74 (10.9)
Body mass index for age									
Underweight	2 (2.3)	5 (4.1)	0 (0.0)	1 (1.4)	0 (0.0)	2 (2.7)	1 (1.0)	1 (1.1)	12 (1.8)
Normal weight	80 (92.0)	111 (90.2)	59 (92.2)	68 (93.2)	62 (89.9)	69 (94.5)	89 (91.8)	88 (93.6)	626 (92.1)
Overweight	5 (5.7)	7 (5.7)	5 (7.8)	4 (5.5)	7 (10.1)	2 (2.7)	7 (7.2)	5 (5.3)	42 (6.2)
Height for age									
Low	5 (5.7)	6 (4.9)	0 (0.0)	9 (12.3)	3 (4.3)	7 (9.6)	13 (13.4)	8 (8.5)	51 (7.5)
Risk of low height	5 (5.7)	9 (7.3)	8 (12.5)	6 (8.2)	11 (15.9)	7 (9.6)	15 (15.5)	7 (7.4)	68 (10.0)
Normal	77 (88.5)	108 (87.8)	56 (87.5)	58 (79.5)	55 (79.7)	59 (80.8)	69 (71.1)	79 (84.0)	561 (82.5)

Overall, the mean EI was 2091.3 kcal/day, while the mean EE was 2067.8 kcal/day, resulting in a small positive EIG of 23.5 kcal/day. Chile had the lowest average for daily EI (mean 1884.6 kcal; 95% CI: 1777.9; 1991.3) among all countries, while Argentina had the highest average for daily EI (mean 2323.1 kcal; 95% CI: 2202.7; 2443.6). The difference between the two countries was 438.5 kcal. For EE, the highest values were in Costa Rica (mean: 2172.2 kcal; 95% CI: 2047.0; 2297.3) and the lowest was in Peru (mean: 1963.7 kcal; 95% CI: 1882.7; 2044.7). The mean difference between the two countries was 208.5 kcal. Regarding the EIG, the mean difference between Argentina (highest energy balance) and Chile (lowest energy balance) was 489.1 kcal (Table 2).

**Table 2:** Energy imbalance gap and its components according to sociodemographic characteristics.

Variables	n (%)	Mean (95% CI) of EI (kcal/day)	Mean (95% CI) of EE (kcal/day)	Mean (95% CI) of EIG (kcal/day)
Overall	680 (100.0)	2091.3 (2054.0;2128.6)	2067.8 (2033.6;2102.1)	23.5 (−16.9;63.8)
Country				
Argentina	87 (12.8)	2323.1 (2202.7;2443.6)	2116.9 (2026.1;2207.8)	206.2 (91.9;320.5)
Brazil	123 (18.1)	2046.1 (1944.1;2148.1)	2070.6 (1982.6;2158.6)	−24.5 (−129.9;80.9)
Chile	64 (9.4)	1884.6 (1777.9;1991.3)	2167.5 (2053.6;2281.4)	−282.9 (−408.8;-156.9)
Colombia	73 (10.7)	2190.9 (2085.9;2296.0)	2020.5 (1927.2;2113.7)	170.5 (59.4;281.5)
Costa Rica	69 (10.1)	2003.8 (1895.3;2112.3)	2172.2 (2047.0;2297.3)	−168.4 (−299.7;-37.0)
Ecuador	73 (10.7)	2177.0 (2081.0;2273.0)	2103.1 (2010.3;2195.9)	74.0 (−33.4;181.3)
Peru	97 (14.3)	2087.1 (2002.1;2172.1)	1963.7 (1882.7;2044.7)	123.4 (30.3;216.5)
Venezuela	94 (13.8)	2001.2 (1906.2;2096.1)	1991.2 (1892.3;2090.1)	10.0 (−96.3;116.2)
Sex				
Male	390 (57.4)	2276.2 (2228.3;2324.0)	2311.3 (2268.8;2353.7)	−35.1 (−93.7;23.5)
Female	290 (42.6)	1842.7 (1796.7;1888.6)	1740.4 (1713.6;1767.3)	102.2 (50.7;153.7)
Socio-economic status				
Low	361 (53.1)	2102.6 (2051.0;2154.1)	2047.3 (2003.2;2091.5)	55.2 (0.3;110.1)
Middle	258 (37.9)	2067.9 (2007.0;2128.8)	2073.7 (2015.6;2131.8)	−5.8 (−72.1;60.5)
High	61 (9.0)	2123.5 (2000.4;2246.5)	2164.2 (2030.5;2297.9)	−40.8 (−180.7;99.2)
Education level				
Basic	563 (82.8)	2113.7 (2071.9;2155.5)	2084.8 (2047.3;2122.3)	28.9 (−16.2;74.1)
High school	98 (14.4)	1966.9 (1880.0;2053.7)	1946.5 (1867.3;2025.6)	20.4 (−71.1;111.9)
Undergraduate	19 (2.8)	2069.2 (1855.8;2282.5)	2191.3 (1867.9;2514.7)	−122.1 (−428.8;184.6)
Ethnicity				
Caucasian	234 (34.4)	2092.6 (2028.8;2156.3)	2127.3 (2060.9;2193.6)	−34.7 (−107.7;38.3)
Mestizo	331 (48.7)	2085.2 (2033.3;2137.1)	2018.2 (1975.1;2061.3)	67.0 (12.6;121.4)
Afro-American	32 (4.7)	2078.8 (1848.4;2309.1)	2009.1 (1856.2;2162.0)	69.7 (−156.4;295.8)
Indigenous	9 (1.3)	2006.9 (1808.8;2204.9)	2163.8 (1840.8;2486.7)	−156.9 (−392.5;78.7)
Others	74 (10.9)	2130.1 (2008.3;2251.9)	2115.5 (2003.2;2227.9)	14.6 (−112.4;141.6)
Body mass index for age				
Underweight	12 (1.8)	2233.5 (1949.6;2517.5)	1887.3 (1736.0;2038.6)	346.2 (70.2;622.2)
Normal weight	626 (92.1)	2086.7 (2048.2;2125.3)	2044.2 (2011.6;2076.7)	42.5 (2.7;82.4)
Overweight	42 (6.2)	2119.0 (1940.2;2297.8)	2472.1 (2231.4;2712.9)	−353.1 (−595.5;-110.7)
Height for age				
Low	51 (7.5)	2029.0 (1900.0;2157.9)	1921.8 (1835.2;2008.4)	107.2 (−10.5;224.8)
Risk of low height	68 (10.0)	2041.3 (1921.6;2160.9)	2000.0 (1891.9;2108.1)	41.3 (−84.7;167.3)
Normal	561 (82.5)	2103.0 (2061.6;2144.4)	2089.3 (2050.8;2127.8)	13.7 (−31.7;59.1)

95% CI, confidence interval 95%; EI, energy intake; EE, energy expenditure; EIG, energy imbalance gap.

In terms of sex differences, males had higher EI and EE than females, resulting in a negative EIG (−35.1 kcal/day) for males and a positive EIG (102.2 kcal/day) for females.

Socioeconomic status did not show significant differences in EIG, although those in the low SES group had a slightly higher EIG than those in the middle or high SES groups. Education level also did not show significant differences in EIG, although those with an undergraduate degree had a slightly larger negative EIG (−122.1 kcal/day) than those with only basic or high school education.

Ethnicity showed some differences in EIG, with Indigenous individuals having the largest negative EIG (−156.9 kcal/day) and Afro-American individuals having a small positive EIG (69.7 kcal/day). Finally, BMI by age showed significant differences in EIG, with underweight individuals having the largest positive EIG (346.2 kcal/day) and overweight individuals having the largest negative EIG (−353.1 kcal/day).

**Figure 2:**
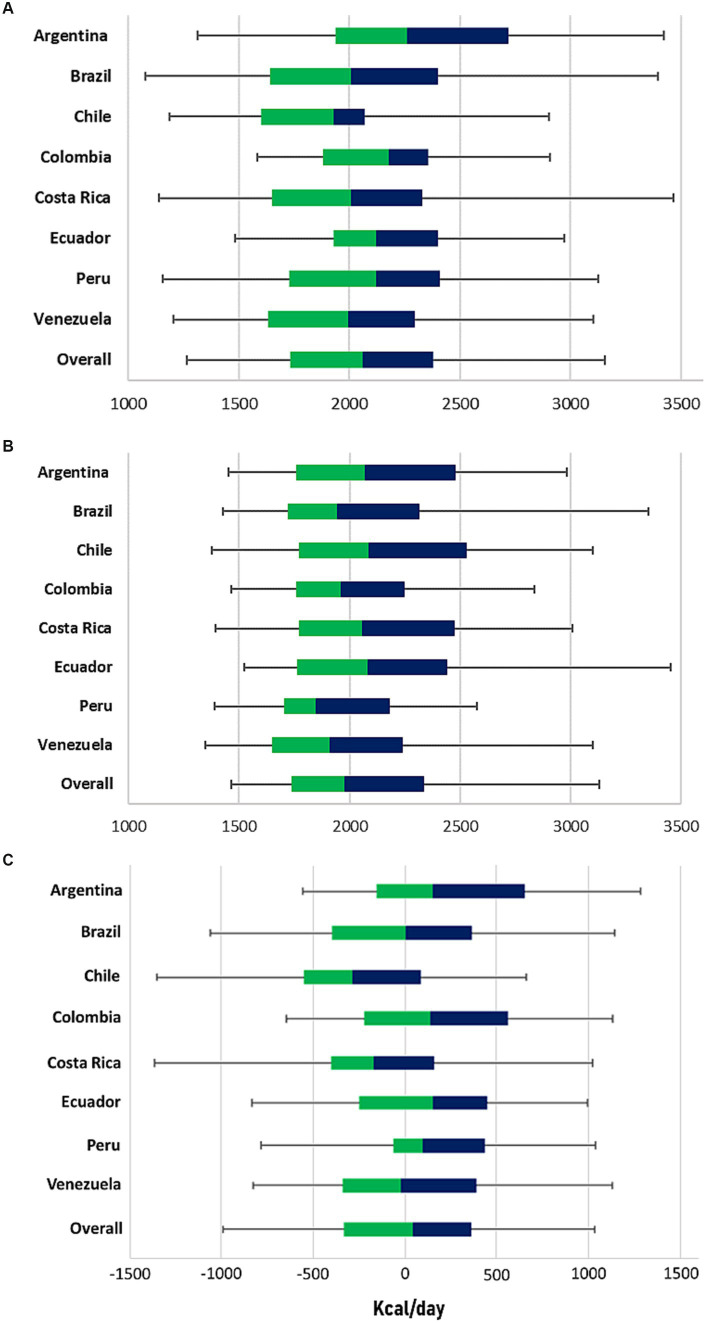
Boxplot of **(A)** energy intake, **(B)** energy expenditure, and **(C)** energy imbalance gap values by country. In the figure, the median is represented by the vertical line between the colors in the box. The part of the box above the median is shown in blue, and the part of the box below the median is shown in green.

Among the EI percentiles (Figure 2A), Argentina consumed the most energy and Chile had the lowest caloric intake (2262.4 kcal and 1930.1 kcal). By sex, all percentiles for males were higher than those for females. In addition, subjects with high SES, underweight subjects, and people with normal height consumed more energy at the 50th percentile (see Supplementary Table S1).

EE (Figure 2B), in Chile was the highest, and Peru was the one with the lowest expenditure (2084.5 kcal and 1848.5 kcal, respectively). In addition, in all countries, males spent more energy than females, as did individuals with high SES, overweight, and normal height (Supplementary Table S2).

In Figure 2C, Ecuador had the highest positive EIG at the 50th percentile, while Chile had the highest negative EIG, with values of 144.9 kcal and − 291.5 kcal, respectively. By sex, males had a negative energy balance at the percentile 50th, and females had a positive balance (−33.4 kcal and 94.0 kcal, respectively). In addition, subjects with high SES had a negative energy balance compared to the rest of the group, as did the overweight subjects. In contrast, in terms of height for age, all categories had a positive EIG at the 50th percentile (Supplementary Table S3).

The associations between the correlates and the total EI, EE, and EIG are presented in Table 3. As expected, the results showed that males consume more energy than females and had a higher EE (433.0 kcal and 581.0 kcal, respectively; *p* < 0.05), therefore they had a negative energy balance in relation to the female sex (−148.1 kcal; *p* < 0.05). In the SES, the subjects with high SES spent more energy than those within low SES (110.1 kcal; *p* < 0.05). Regarding the BMI for age, it was observed that underweight adolescents and normal-weight subjects spent less energy than overweight subjects (*p* < 0.05), so these two groups had a positive energy balance (+979.6 kcal and + 394.1 kcal, respectively). Finally, in terms of height for age, the risk of low-and low-height subjects consumed and spent less energy than normal height subjects, but they had a positive energy balance; however, these results were not significant (*p* > 0.05).

**Table 3:** Adjusted multilevel linear regression models [b coefficient (95% CI)] for the relationships between independent variables and energy intake, energy expenditure and energy imbalance gap.

Independent variables	Energy intake (kcal/day)	Energy expenditure (kcal/day)	Energy imbalance gap (kcal/day)
Sex			
Female^1^	Ref.	Ref.	Ref.
Male	433.0 (364.6; 501.4)	581.0 (529.8; 632.3)	−148.1 (−227.7; −68.4)
Socioeconomic status			
Low^2^	Ref.	Ref.	Ref.
Middle	−31.5 (−104.3; 41.3)	16.5 (−37.1; 70.2)	−48.0 (−132.2; 36.1)
High	20.1 (−104.0; 144.2)	110.1 (22.4; 197.8)	−90.0 (−230.3; 50.3)
Body mass index for age			
Overweight^3^	Ref.	Ref.	Ref.
Normal weight	−40.4 (−180.7; 100.0)	−434.5 (−539.6; −329.3)	394.1 (230.4; 557.8)
Underweight	−50.1 (−419.9; 319.7)	−1029.7 (−1319.0; −740.5)	979.6 (518.6; 1440.6)
Height for age			
Normal^4^	Ref.	Ref.	Ref.
Risk of low height	−22.9 (−136.1; 90.3)	−40.9 (−125.6; 43.8)	18.0 (−115.3; 151.3)
Low	−41.1 (−171.1; 88.9)	−95.4 (−192.4; 1.6)	54.3 (−97.2; 205.7)

Adjusted for ^1^sex, ^2^socio-economic status, ^3^body mass index, and ^4^height.

**Figure 3:**
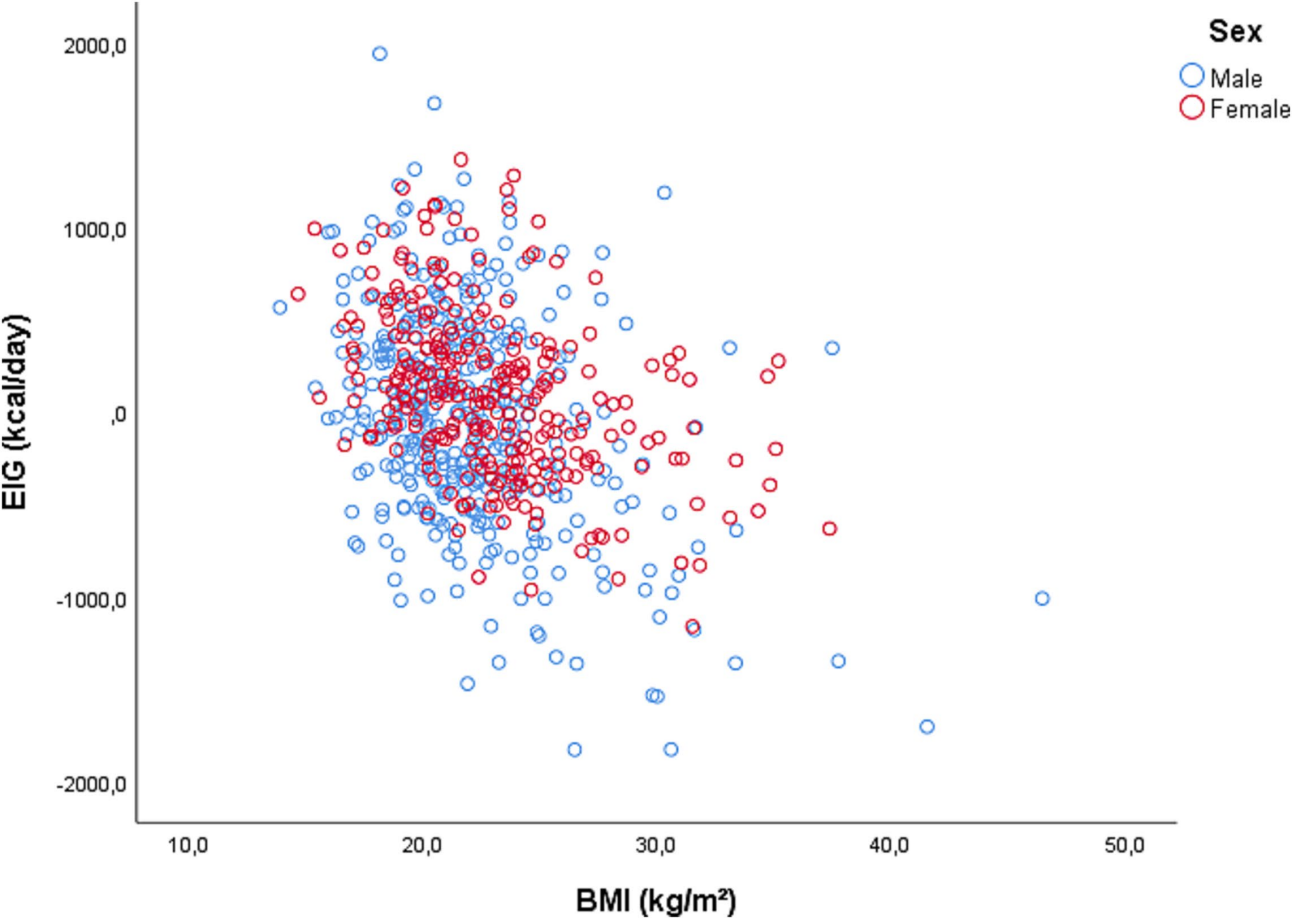
Scatter-dot plot of the energy imbalance gap as a function of body mass index in the study participants.

Figure 3 shows a Scatter-dot plot of the energy imbalance gap as a function of body mass index in the study participants. The results showed a significant negative correlation between BMI and EIG (*r* = −0.40, *p* < 0.001), indicating that as BMI increased, EIG decreased. This suggests that individuals with normal BMI or overweight tend to have a smaller energy imbalance gap, potentially contributing to weight gain or maintenance. Furthermore, the analysis revealed a greater dispersion of EIG in men compared to women.

## 4 Discussion

This study examined first the gaps between EI and EE, and second, the associations between EIG and sociodemographic and nutritional status variables in the urban adolescent population of ELANS countries. The results showed that there was a positive energy balance in general, with EI values (2091 kcal) being higher than EE values (2068 kcal).

Growth and development have a significant impact on the energy requirements during adolescence. Adolescents typically need extra calories to fuel metabolic and physical activities, as well as to increase the amount of important body tissues such as bone, muscle, blood, and body organs (35). A positive energy balance is necessary for growth; however, it is also important for adolescents to maintain a healthy energy balance and avoid weight gain. A negative energy balance can prevent adolescents from growing properly, which is a public health concern (36).

The WHO (37) has strengthened its approach toward adolescence as a period in which rapid changes are manifested, and risks for diseases need to be addressed. According to the same source, adolescents between 10 and 14 years old are more at risk of experiencing diseases as a result of lack of sanitation; however, the risks associated with those between 15 and 19 years old are related to behaviors including diet, PA, substance misuse, and unsafe sex.

The positive EIG in most ELANS countries suggests that adolescents consume more energy than they expend, which could lead to weight gain over time. This is especially concerning in Argentina, Colombia, and Peru, where the EIG is higher than 100 calories. Previous studies have found that these three countries also have a positive energy balance in their adult populations (5). Additionally, a study of the anthropometric profiles of ELANS countries found that Argentina had the fourth highest prevalence of obesity (38).

In terms of the distribution of macronutrients, which are the nutrients that provide energy and constitute the EI, a previous ELANS study (39) found that in adolescents the 54.9% of their calories came from carbohydrates, 29.7% from lipids, and 15.4% from proteins. These percentages were consistent across different age groups and sexes. Peru had the highest percentage of energy derived from carbohydrates (62.9%), whereas Argentina and Colombia had the highest percentage of energy derived from fats (32.6 and 30.8%, respectively).

Argentina is a significant contributor to global beef production, accounting for approximately 5% of the total global output, and is one of the countries with the highest *per capita* meat consumption (40). In fact, animal-based proteins account for nearly 70% of the total daily protein intake (41). Additionally, it was the country with the highest processed food such as soft drinks, cookies and crackers, pizza, sugar and sweets, processed beef, cakes, salad dressing, and ice cream, many of which are sources of fat, in parallel it was country with the lowest consumption of fruits, legumes, rice, roots, and fish (39).

In Colombia, fat intake is mainly due to the use of oils for frying and the consumption of ultra-processed food products (42). The food groups that contributed the most to EI were fats, dairy products, eggs, nuts and seeds, roots, and alcoholic beverages, while the grains, pasta, bread, and soda drinks were the lowest contributors (39).

The agricultural industry in Peru has shown significant growth over the past decades, making it the second-largest economic sector in the country (43); thus, fresh food products are important in the Peruvian diet. In this case, grains, bread, pasta, potatoes, whole grain products, fruits, and vegetables were the principal contributors to the total EI (39), whereas poultry was the main protein source (41).

In terms of energy expenditure, Peru had the lowest value in this study, probably because of its low level of PA. A self-reported survey conducted in six South American countries showed that the highest levels of leisure physical inactivity (< 150 min/week) were in Peru (91.4%). A previous ELANS experience (44) noted that Peruvian adolescents and youth adults had the highest sitting time (556.8 min/day), which could explain their low EE. Wei et al. (45) described a model that suggests that Hispanic adolescents are likely to participate in less PA than others, which gives them a higher risk of obesity.

The higher EI and EE in male adolescents than in females was consistent with the results of previous studies. Silva et al. (46) studied 459 adolescents aged 10 to 17 in Portugal and found an energy imbalance gap of −50.7 kcal in males, with both EI and EE significantly higher than in females. Literature has consistently supported that male adolescents have a higher basal metabolic rate (BMR) and are more active than females (47, 48). Males have a greater BMR per unit of body than females because of body composition differences, including lean body mass (47). Additionally, males spend more time on high-intensity activities than females who spend more time on low-intensity activities (48). These findings suggest that biological and behavioral factors contribute to higher EI and EE in male adolescents.

The EE reported by adolescents in the present study was higher in subjects with a high SES than in those with a low SES. This is likely because adolescents from higher SES families are more likely to live in neighborhoods with parks, playgrounds, and other facilities that are conducive to PA, normally have the financial resources to purchase sports equipment and transportation for sports activities (45, 49), and are more likely to receive encouragement and support from their parents and peers to be physically active (50, 51). However, further studies are necessary to confirm this finding in the urban areas of Latin American.

Regarding BMI, there was a positive EIG in underweight and normal-weight adolescents compared to overweight adolescents. This is likely due to the compensatory strategies. Underweight adolescents had the lowest EE, which is likely due to a compensatory decrease in PA and a lower BMR due to their lower body weight. It is well established that underweight can induce reductions in EE and slow growth to favor subsequent body weight gain (52). Lazzer et al. (53) found that obese adolescents have a higher BMR due to their high weight, and higher EE compared to non-obese subjects. They also found that obese subjects spent more time on light physical activities, such as shopping or slow walking during the weekend, but much less time on moderate and sports activities than non-obese subjects. This could explain the results of Chile and Costa Rica, which had the lowest EIG values, with a higher EE than EI and a high prevalence of obesity by BMI (38). Therefore, despite a negative energy balance, they do not present high levels of malnutrition in their population. Thivel et al. (54) established that obese adolescents may spontaneously decrease EI after intensive exercise, an important compensatory theory, especially for this research because overweight adolescents had an EI very similar to that of the normal weight group.

Burns (55), also revealed a discrepancy in the correlations between weight loss intent and the practice of specific energy balance-related health behaviors in adolescents. A cross-sectional study found that less movement does not necessarily imply lower energy expenditure (56). Thivel et al. (54) reported that energy intake (EI) in today’s obesogenic environment may primarily be determined by non-homeostatic pathways that can override the body’s energy and hormonal signals. This is because EI changes are not influenced by the amount of energy expended during activities, such as screen-based sedentary behaviors, which are more likely to stimulate food intake (regardless of appetite sensations) than non-screen sedentary behaviors. Therefore, accurately measuring energy balance and body changes remains challenging.

This study’s innovative findings include identifying sex and BMI as factors associated with EIG among adolescents from Latin America. The inverse relationship between BMI and EIG is an interesting finding that should be studied in depth in the future. The study also highlights the variability in EIG across different countries in the region.

The authors’ previous experience addressed the relationships between sociodemographic variables and nutritional status with EIG in LA adults. Nevertheless, this study has several strengths, including its focus on adolescent EIG, for which research is scarce. We also have used two non-consecutive 24-h recalls, the PA recall using the extensively validated IPAQ questionnaire, and the randomized multistage sampling. Additionally, a multilevel linear regression model was used to examine the individual and contextual factors that may contribute to the energy imbalance gap, providing a more comprehensive understanding of the issue.

However, this study has some limitations that need to be considered. First, this study was cross-sectional; therefore, it could not be used to establish causality. The data on EI and EE were self-reported, which may have introduced bias in the technique used. This study was conducted in a limited number of countries; therefore, the findings may not be generalizable to all LA regions. Moreover, BMI does not differentiate between excess fat, muscle, or bone mass, nor does it offer any insight into fat distribution in individuals. Longitudinal studies are needed to better understand how changes in BMI and EIG over time impact energy balance and weight management. Addressing these limitations in future research could enhance the understanding of the relationship between BMI and EIG, as well as gender-specific differences in energy balance regulation among adults.

On the other hand, the methodology used in this study to evaluate EIG has been controversially discussed as it goes away from direct and indirect calorimetry using a metabolic chamber and metabolic cart, respectively, and the doubly labeled water technique (57). However, these methods are costly or invasive to perform in epidemiological studies. Thus, the results of this study constitute a first step toward understanding the relationship between EI and EE in LA adolescents, and may constitute an input to promote public policies aimed at reducing the prevalence of obesity in urban areas of the LA region.

## 5 Conclusion

In conclusion, the findings of this study suggest that sex and BMI are associated with EIG among adolescents in ELANS countries. These factors should be considered when developing public health policies to prevent weight gain and promote healthy weight in this age group. The findings of this study also suggest first actions directed to raise awareness on the EIG research in epidemiologic studies, and allocate funds to provide the resources to evaluate at least some neuro-endocrine markers that are involved in the mechanisms of energy intake, as usually funds are an important obstacle for not including those type of variables in an epidemiologic study. Second, policy makers and stake holders need to be aware that adolescents are a fundamental key pillar of societal wellbeing as is it during this life phase that future health and fertility are consolidated, and the window of opportunities for interventions during the period of growth and development goes to an end, therefore there is a need to implement public health interventions to address the issue of EIG in adolescents in LA, that translate into better health care actions, including screening, promoting healthy eating habits and better built environments less obesogenic and friendly for the practice of daily life physical activities.

## Data availability statement

The raw data supporting the conclusions of this article will be made available by the authors, without undue reservation.

## Ethics statement

The study was conducted in accordance with the Declaration of Helsinki. Ethical approval was provided by the Western Institutional Review Board (#20140605), and by the ethical review boards of the participating institutions. This study is registered at Clinical Trials #NCT02226627. Written informed consent/assent was obtained from all individuals before commencement of the study.

## Author contributions

PH: Conceptualization, Visualization, Writing – original draft. MH-C: Data curation, Investigation, Methodology, Resources, Supervision, Writing – original draft. GF: Formal analysis, Writing – review & editing. RY: Formal analysis, Writing – review & editing. MY: Conceptualization, Data curation, Investigation, Methodology, Resources, Writing – original draft. MV: Methodology, Writing – original draft. LC: Conceptualization, Investigation, Methodology, Resources, Writing – original draft. YS: Investigation, Resources, Supervision, Writing – original draft. ML-J: Investigation, Resources, Supervision, Writing – original draft. GG: Data curation, Investigation, Resources, Writing – review & editing. RM-R: Data curation, Investigation, Resources, Writing – review & editing. RP: Data curation, Investigation, Resources, Writing – review & editing. AR: Data curation, Investigation, Resources, Writing – review & editing. IK: Data curation, Funding acquisition, Investigation, Resources, Writing – review & editing. MF: Data curation, Funding acquisition, Investigation, Resources, Writing – review & editing.

## ELANS study group

Chairs: Mauro Fisberg and Irina Kovalskys; Co-chair: Georgina Gómez; Core Group members: Attilio Rigotti, Lilia Yadira Cortés, Georgina Gómez, Martha Cecilia Yépez García, Rossina Gabriela Pareja, and Marianella Herrera-Cuenca; Project Managers: Viviana Guajardo and Ioná Zalcman Zimberg; Dietary Intake Advisor: Agatha Nogueira Previdelli; Physical Activity Advisor: Gerson Ferrari. In addition, the authors would like to thank the external committee, Berthold Koletzko, Luis A. Moreno, and Miichael Pratt.
