# Supplementary material for: Energy imbalance gap was associated with body mass index and sex in Latin American adolescents—results from the ELANS study
Source: Front Nutr. 2024 Mar 14;11:1380315. doi: 10.3389/fnut.2024.1380315 (PMC10972923; doi:10.3389/fnut.2024.1380315)
Supplement: Supplementary file 1 [file Table_1.DOCX]

Supplementary Material

**Energy imbalance gap was associated with body mass index and sex in Latin American adolescents—Results from the ELANS Study**

**Supplementary Table S1.** Energy intake percentiles by sociodemographic variables.

|  | **Energy Intake Percentiles (kcal/day)** | | | | | | |
| --- | --- | --- | --- | --- | --- | --- | --- |
|  | **3** | **10** | **25** | **50** | **75** | **90** | **97** |
| **Country** |  |  |  |  |  |  |  |
| Argentina | 1314.68 | 1618.74 | 1937.78 | 2262.40 | 2719.37 | 3120.57 | 3422.21 |
| Brazil | 1079.77 | 1360.31 | 1640.60 | 2008.97 | 2399.83 | 2785.79 | 3393.99 |
| Chile | 1187.70 | 1367.82 | 1601.46 | 1930.11 | 2067.78 | 2396.42 | 2901.50 |
| Colombia | 1368.50 | 1631.12 | 1880.24 | 2179.02 | 2356.96 | 2690.30 | 3490.05 |
| Costa Rica | 1204.67 | 1453.49 | 1649.77 | 2008.02 | 2332.41 | 2608.45 | 2903.05 |
| Ecuador | 1360.00 | 1662.23 | 1930.87 | 2121.37 | 2399.74 | 2767.76 | 3116.60 |
| Peru | 1430.79 | 1549.60 | 1726.62 | 2121.12 | 2407.82 | 2625.12 | 2956.56 |
| Venezuela | 1206.24 | 1382.10 | 1634.18 | 1993.65 | 2296.01 | 2575.38 | 3104.36 |
| Overall | 1266.10 | 1475.34 | 1731.75 | 2061.76 | 2377.98 | 2719.37 | 3153.91 |
| **Gender** |  |  |  |  |  |  |  |
| Male | 1399.96 | 1675.88 | 1972.42 | 2225.20 | 2584.03 | 2898.58 | 3321.62 |
| Female | 1192.44 | 1363.61 | 1562.15 | 1810.04 | 2071.40 | 2341.85 | 2825.43 |
| **Socio-economic level** |  |  |  |  |  |  |  |
| Low | 1220.19 | 1493.40 | 1731.81 | 2071.32 | 2396.73 | 2737.87 | 3118.30 |
| Middle | 1254.73 | 1445.10 | 1717.49 | 2023.18 | 2326.11 | 2716.32 | 3194.06 |
| High | 1267.95 | 1462.62 | 1763.38 | 2106.34 | 2475.71 | 2798.48 | 3071.77 |
| **Body mass index for age** |  |  |  |  |  |  |  |
| Underweight | 1622.00 | 1650.63 | 1794.81 | 2195.34 | 2632.83 | 2916.98 | 3215.98 |
| Normal weight | 1277.07 | 1474.54 | 1731.10 | 2058.38 | 2355.77 | 2719.85 | 3151.84 |
| Overweight | 1108.26 | 1396.08 | 1698.48 | 2122.59 | 2414.95 | 2805.79 | 3683.46 |
| **Height for age** |  |  |  |  |  |  |  |
| Low | 1132.29 | 1463.02 | 1697.96 | 2037.98 | 2308.13 | 2699.50 | 3020.46 |
| Risk of low height | 1092.19 | 1361.11 | 1645.42 | 2063.74 | 2323.11 | 2719.19 | 3192.59 |
| Normal | 1309.51 | 1475.85 | 1739.44 | 2070.72 | 2395.66 | 2736.56 | 3158.03 |

**Supplementary Table S2**. Energy expenditure percentiles by sociodemographic variables.

|  | **Energy Expenditure Percentiles (kcal/day)** | | | | | | |
| --- | --- | --- | --- | --- | --- | --- | --- |
|  | **3** | **10** | **25** | **50** | **75** | **90** | **97** |
| **Country** |  |  |  |  |  |  |  |
| Argentina | 1489.60 | 1561.61 | 1758.40 | 2069.44 | 2478.95 | 2702.29 | 2985.11 |
| Brazil | 1417.94 | 1576.65 | 1719.29 | 1943.51 | 2316.11 | 2801.77 | 3350.83 |
| Chile | 1457.32 | 1601.70 | 1772.48 | 2084.47 | 2530.10 | 2798.98 | 3102.53 |
| Colombia | 1380.67 | 1577.81 | 1758.80 | 1960.79 | 2247.84 | 2695.75 | 2782.51 |
| Costa Rica | 1477.08 | 1604.33 | 1772.15 | 2055.56 | 2475.64 | 2969.20 | 3489.15 |
| Ecuador | 1368.58 | 1588.56 | 1762.87 | 2080.68 | 2440.36 | 2611.26 | 2833.95 |
| Peru | 1466.38 | 1521.31 | 1704.67 | 1848.47 | 2183.59 | 2557.88 | 2773.70 |
| Venezuela | 1358.17 | 1500.19 | 1648.39 | 1907.67 | 2241.99 | 2530.89 | 3099.39 |
| Overall | 1429.02 | 1561.61 | 1735.61 | 1977.41 | 2336.07 | 2696.30 | 3130.23 |
| **Gender** |  |  |  |  |  |  |  |
| Male | 1708.64 | 1818.93 | 1981.64 | 2223.91 | 2566.72 | 2830.18 | 3315.33 |
| Female | 1372.54 | 1473.34 | 1568.21 | 1712.66 | 1875.18 | 2054.76 | 2251.23 |
| **Socioeconomic level** |  |  |  |  |  |  |  |
| Low | 1443.81 | 1560.18 | 1727.42 | 1977.64 | 2277.33 | 2639.10 | 2917.95 |
| Middle | 1423.01 | 1557.94 | 1738.99 | 1963.82 | 2355.69 | 2709.10 | 3274.25 |
| High | 1518.41 | 1579.61 | 1743.20 | 2034.63 | 2506.18 | 2824.99 | 3516.60 |
| **Body mass index for age** |  |  |  |  |  |  |  |
| Underweight | 1620.64 | 1628.95 | 1676.59 | 1781.98 | 2134.10 | 2241.38 | 2455.94 |
| Normal weight | 1426.27 | 1559.20 | 1735.75 | 1970.86 | 2306.63 | 2648.41 | 2852.28 |
| Overweight | 1443.53 | 1569.48 | 1767.84 | 2408.56 | 3315.11 | 3501.40 | 4049.00 |
| **Height for age** |  |  |  |  |  |  |  |
| Low | 1463.32 | 1563.44 | 1709.61 | 1835.21 | 2146.00 | 2419.90 | 2650.22 |
| Risk of low height | 1377.52 | 1533.38 | 1685.64 | 1907.41 | 2204.00 | 2636.92 | 3322.52 |
| Normal | 1426.30 | 1561.61 | 1745.96 | 2011.38 | 2381.55 | 2719.17 | 3149.35 |

**Supplementary Table S3**. Energy imbalance gap percentiles by sociodemographic variables.

|  | **Energy Imbalance Gap Percentiles (kcal/day)** | | | | | | |
| --- | --- | --- | --- | --- | --- | --- | --- |
|  | **3** | **10** | **25** | **50** | **75** | **90** | **97** |
| **Country** |  |  |  |  |  |  |  |
| Argentina | -554.63 | -410.97 | -197.71 | 132.26 | 612.46 | 975.32 | 1294.52 |
| Brazil | -1052.17 | -772.11 | -399.98 | -7.07 | 349.72 | 664.77 | 1114.69 |
| Chile | -1361.27 | -933.01 | -559.35 | -291.53 | 83.93 | 333.57 | 662.86 |
| Colombia | -647.63 | -495.85 | -215.12 | 126.28 | 559.10 | 810.06 | 1127.92 |
| Costa Rica | -1361.43 | -944.47 | -402.38 | -167.74 | 163.95 | 502.96 | 1029.14 |
| Ecuador | -833.11 | -521.09 | -271.30 | 144.91 | 410.55 | 610.04 | 996.99 |
| Peru | -783.56 | -326.67 | -79.63 | 85.41 | 429.15 | 800.26 | 1031.29 |
| Venezuela | -823.93 | -622.98 | -338.68 | -19.32 | 387.12 | 688.23 | 1124.22 |
| Overall | -1009.37 | -613.55 | -316.32 | 35.56 | 354.38 | 710.72 | 1057.13 |
| **Gender** |  |  |  |  |  |  |  |
| Male | -1242.68 | -757.01 | -391.30 | -33.43 | 368.76 | 710.31 | 1098.45 |
| Female | -679.66 | -453.35 | -206.16 | 94.01 | 347.96 | 718.70 | 1052.10 |
| **Socio-economic level** |  |  |  |  |  |  |  |
| Low | -912.71 | -585.76 | -300.60 | 35.89 | 388.60 | 776.60 | 1101.88 |
| Middle | -1171.22 | -631.84 | -354.28 | 40.89 | 340.68 | 623.78 | 1097.81 |
| High | -1215.31 | -880.27 | -300.33 | -12.25 | 394.57 | 637.31 | 957.41 |
| **Body mass index for age** |  |  |  |  |  |  |  |
| Underweight | -332.67 | -269.56 | -32.03 | 346.53 | 686.65 | 977.99 | 1360.67 |
| Normal weight | -894.67 | -571.71 | -298.31 | 46.78 | 353.95 | 697.90 | 1070.01 |
| Overweight | -1790.94 | -1480.80 | -967.13 | -361.94 | 328.32 | 769.70 | 1098.58 |
| **Height for age** |  |  |  |  |  |  |  |
| Low | -713.53 | -538.41 | -80.37 | 115.60 | 395.36 | 682.36 | 945.24 |
| Risk of low height | -1158.02 | -554.55 | -290.65 | 59.67 | 356.66 | 816.28 | 1027.04 |
| Normal | -1011.51 | -637.64 | -326.03 | 1.41 | 354.16 | 716.06 | 1110.12 |
